# Influência da Gordura do Braço sobre Medida Indireta da Pressão Sanguínea: Uma Abordagem Estatística e de *Machine Learning*

**DOI:** 10.36660/abc.20220484

**Published:** 2023-05-05

**Authors:** Paôla de Oliveira Souza, José Maria Parente de Oliveira, Letícia Helena Januário

**Affiliations:** 1 Instituto Federal de Minas Gerais Ibirité MG Brasil Instituto Federal de Minas Gerais, Ibirité, MG – Brasil; 2 Instituto Tecnológico de Aeronáutica São José dos Campos SP Brasil Instituto Tecnológico de Aeronáutica, São José dos Campos, SP – Brasil; 3 Universidade Federal de São João del-Rei Divinópolis SP Brasil Universidade Federal de São João del-Rei, Divinópolis, SP – Brasil

**Keywords:** Pressão arterial, Análise de Regressão, Análise por Conglomerados, Braço

## Abstract

**Fundamento:**

A mensuração indireta da pressão sanguínea (PS) é sabidamente influenciada por diversos fatores como técnica, observador e equipamento, mas a influência da composição do braço ainda não foi investigada.

**Objetivo:**

Identificar a influência da gordura do braço sobre a medida indireta da pressão sanguínea, utilizando modelos de inferência estatística e *machine learning*.

**Métodos:**

Estudo transversal, com 489 adultos jovens saudáveis de 18 a 29 anos de idade. Foram medidos comprimento (COB), circunferência do braço (CB) e índice de gordura do braço (IGB). A PS foi mensurada em ambos os braços, simultaneamente. Os dados foram processados utilizando-se Python 3.0 e pacotes específicos para análise descritiva, regressão e clusterização. Um nível de significância de 5% foi adotado para todos os cálculos.

**Resultados:**

A PS e as medidas antropométricas foram diferentes entre os hemicorpos. A pressão sanguínea sistólica (PAS), COB, e IGB foram maiores no braço direito (BD), enquanto CB foi similar em comparação ao braço esquerdo. COB e CB apresentaram correlação positiva com a PAS. Conforme o modelo de regressão, para determinado valor de CB e COB, a leitura da PAS poderá ter uma redução média de 1,80 mmHg no BD, e 1,62 mmHg no braço esquerdo, a cada 10% de aumento na IGB. A clusterização corroborou os resultados da regressão.

**Conclusão:**

Foi encontrada uma influência significativa do IGB sobre a leitura da PS. A PAS teve correlação positiva com COB e CB, e correlação negativa com IGB, o que sugere a necessidade de mais investigações sobre a relação da PS com as frações de gordura e músculo do braço.

## Introdução

A medição precisa da pressão sanguínea (PS) é fundamental para o diagnóstico e tratamento adequado da hipertensão arterial sistêmica (HAS). Como os portadores de HAS habitualmente não apresentam sintomas, o diagnóstico é realizado nas consultas de rotina em consultórios médicos e/ou nos serviços de atenção básica de saúde. Apesar do grande avanço tecnológico para tratamento das lesões nos órgãos alvo, a identificação e a classificação da HAS, bem como o limiar e a meta para o tratamento, ainda se baseiam na medida indireta da pressão sanguínea (MIPS).^1^

A padronização da técnica de MIPS, recomendada pelos diversos *guidelines*, destaca a seleção de manguitos, proporcionais à circunferência do braço (CB).^2-8^ Entretanto, em um mesmo valor de CB, há diferentes frações de gordura e músculo. Essas estruturas corporais apresentam diferentes densidades e espessuras, podendo exigir maior ou menor pressão sobre o membro para a compressão da artéria, e se constituir um importante fator de imprecisão na MIPS. Erros e inexatidões deste procedimento podem levar indivíduos a uma terapia de drogas desnecessária ou à falta de tratamento imprescindível.^9^

A possível imprecisão da MIPS em função dos diferentes percentuais de gordura e músculo do braço em indivíduos com a mesma CB não foi encontrada na literatura específica consultada. A identificação dessa variável poderá resultar na estimação mais fidedigna dos valores da PS por métodos indiretos.

Por outro lado, a análise de dados na área da saúde combinando a estatística tradicional com técnicas de *machine learning* (ML) cresce constantemente. O uso de ML apresenta vantagens importantes no desempenho preditivo, na descoberta de propriedades de um conjunto de dados e possíveis correlações de diferentes atributos desse conjunto e na identificação de subpopulações não descobertas e prognósticos específicos.^10,11^

O objetivo deste estudo foi identificar a influência da gordura do braço sobre a MIPS, em adultos jovens saudáveis, utilizando modelos de inferência estatística e ML em uma abordagem de clusterização.

A medida da PS é um procedimento amplamente difundido e realizado por diversos profissionais da área da saúde e também por pessoas leigas. Por isso, a prática pode ser caracterizada como uma prática “banal” e ser executada com desleixo e/ou com maus hábitos aprendidos no início do treinamento profissional.^12^ Entretanto, a acurácia do procedimento demanda competências específicas e atenção às minúcias, o que não ocorre com frequência, mesmo quando realizado por profissionais.^1,13^

A mensuração da PS habitualmente é realizada por um método indireto. A técnica orientada, pelos principais *guidelines* internacionais e diretrizes nacionais, requer cuidados em relação à pessoa cuja PS está sendo medida, ao profissional, ao ambiente e aos equipamentos a serem utilizados.^2,5,7,8,14-19^Entre as recomendações, as dimensões do manguito adequado devem corresponder em comprimento a 100% (mínimo de 80%), e em altura, a 40% da CB.^7,14,17,18^O uso de um manguito maior que o ideal resultará em valores de PS menores que o real, assim como o uso de manguito menor que o ideal resultará em valores da PS maiores que o real.^9,20^

O método auscultatório com manômetros aneróides ainda é muito utilizado no Brasil, embora se observe o crescente uso de equipamentos oscilométricos automatizados. A utilização dos equipamentos automáticos contribui com a redução de erros relacionados à observação do profissional.^21^Entretanto, independentemente dos equipamentos utilizados, a medida da PS é um procedimento determinante para o diagnóstico da HAS, com grande margem de imprecisão.

## Material e Métodos

Trata-se de um estudo descritivo, analítico, de corte transversal, cujos os dados foram coletados na Universidade Federal de São João Del Rei em Divinópolis, Brasil. A população do estudo foi constituída de estudantes da graduação de 18 a 29 anos de idade. Trata-se de uma amostra de conveniência justificada pela restrição de recursos financeiros e pela diversificação das características geoeconômicas da população de adultos jovens saudáveis da universidade em questão. Os dados foram coletados no período de agosto de 2017 a janeiro de 2018, por meio de questionários e medidas de dobras cutâneas de tríceps (DCT), CB, comprimento do braço (COB) e PS.

### Medidas antropométricas

As DCT foram aferidas em milímetros, a CB e COB em centímetros em ambos os braços. Todas as aferições foram feitas com equipamentos certificados, validados e devidamente calibrados e conforme técnicas orientadas pela NHANES.^22^ Foram coletadas três medidas de cada variável e para a análise dos dados foi utilizada a média simples das três.

A partir das medidas antropométricas foi calculado o Índice de Gordura do Braço (IGB), conforme Frisancho,^23^ pela equação:


$$\text{ IGB }=\frac{AGB}{ATB}\times 100$$

Onde AGB é área de gordura do braço (cm^2^) e ATB é área total do braço (cm^2^) cujas fórmulas são, respectivamente:^23^


$$\begin{array}{c} AGB=\frac{2\times DCT\times CB-\pi DCT^{2}}{4}\\ ATB=\frac{CB^{2}}{4\pi } \end{array}$$

### Pressão sanguínea

A mensuração da PS atendeu, rigorosamente, aos *guidelines* nacionais e internacionais.^1,4^ As medidas ocorreram em ambiente tranquilo, descontraído, claro e com privacidade. Os pesquisadores buscaram estabelecer uma relação interpessoal efetiva por meio de atitudes e posturas acolhedoras, conforme recomendado por Kohlmann e Kohlmann.^24^ Foi certificado que o participante não havia fumado, praticado exercícios físicos, ingerido bebidas alcoólicas, café ou alimentos nos últimos 60 minutos e solicitado que esvaziasse a bexiga antes das medidas. O tempo de repouso antes da medida foi de, no mínimo, 10 minutos. O participante permaneceu sentado em poltrona confortável, com ajuste de altura e apoio dos braços e pés, com as pernas descruzadas, o dorso recostado e relaxado e instruído a não conversar. Os braços foram posicionados na altura do coração, com a palma da mão voltada para cima. Foram utilizados manguitos cônicos, adequados à CB e posicionados de 2 a 3 cm acima da fossa cubital e a parte compressiva da braçadeira centralizada sobre a artéria braquial.^1,18^Foram realizadas três medidas nos dois braços simultaneamente, com intervalo de um minuto, com aparelho eletrônico oscilométrico certificado, validado e devidamente calibrado da marca Omron. A simultaneidade foi garantida pela filmagem dos *displays.* Para análise, foi utilizada a média das duas últimas medidas, conforme Leung.^18^

### Análises dos dados

Os dados foram importados e administrados no ambiente JupyterLab (versão 2.2.6) e analisados utilizando-se a linguagem Python 3.0 e os pacotes Pandas, Numpy, MatplotLib, Statsmodels, Seaborn, Plotnine, Sci-Py and Sklearn. O conjunto de dados usados e os notebooks com os procedimentos e códigos (análise descritiva, regressão e clusterização) estão disponíveis para outros pesquisadores para fins de conferência e reprodução dos resultados em um repositório no GitHub (https://github.com/paolaosouza/blood_preassure_analysis).

### Análise estatística

Os dados foram organizados em uma tabela da qual foram selecionadas as variáveis que apresentaram correlação estatisticamente significativas com a pressão arterial sistólica (PAS) e/ou com a pressão arterial diastólica (PAD): COB, CB, DCT e IGB. Uma coluna de intervalos de circunferência foi criada a partir da Regra de Doane. Os dados também foram divididos em duas outras tabelas, uma com as medidas de braço direito e outra com as medidas de braço esquerdo.

O nível de significância foi definido como 5% (p-valor < 0,05) para todos os testes da análise descritiva. O teste de Shapiro-Wilk foi escolhido para verificar se as distribuições das variáveis contínuas se aproximam da normalidade. Para as variáveis contínuas com distribuição normal, utilizou-se da média e desvio-padrão; para as variáveis com distribuição não normal utilizou-se da mediana e intervalos interquartis. As médias das variáveis contínuas com distribuição normal foram comparadas por hemicorpos utilizando-se teste T pareado e as medianas pelo teste não paramétrico de Wilcoxon, dado que os grupos são dependentes. A associação entre as variáveis contínuas foi computada por meio do coeficiente de correlação de Spearman (r).

Um modelo de regressão linear múltipla foi ajustado para cada hemicorpo para se fazer inferência sobre a relação entre as covariáveis e a PAS. Não foi ajustado um modelo de regressão para a PAD, pois não houve evidência de associação com as covariáveis. As variáveis altamente correlacionadas foram descartadas para manter o modelo parcimonioso e evitar multicolinearidade, dando-se prioridade para variáveis compatíveis com o objetivo do estudo. Em seguida, um modelo foi ajustado para cada hemicorpo e outro para os dados de ambos os braços considerando a variável braço como efeito aleatório. A qualidade do ajuste foi avaliada via Critério de Informação Akaike (AIC) e os modelos foram validados pela análise dos resíduos. O pressuposto de normalidade foi validado com um gráfico de quantil e com o teste de Shapiro-Wilk; a independência entre os resíduos com um gráfico de ACF e estatística de Durbin-Watson; e a homocedasticidade através de técnicas gráficas, visualizando-se os resíduos versus covariáveis.

### Análise por *machine learning*

Modelos de ML foram utilizados para acrescentar informações importantes às análises estatísticas realizadas.^9^ Foi escolhida uma abordagem não supervisionada de clusterização para explorar os dados de forma livre de hipóteses e tentar descobrir padrões subjacentes relacionados à pressão sanguínea e medidas antropométricas. O algoritmo de clusterização K-means foi escolhido porque é relativamente simples, computacionalmente rápido de implementar e apresenta resultados razoáveis para a maioria dos problemas.^10^

O algoritmo K-means foi executado para o grupo de dados de braço esquerdo e braço direito separadamente. Inicialmente os valores de todos os atributos foram normalizados para que ficassem no mesmo intervalo; caso contrário o algoritmo de agrupamento poderia acabar priorizando somente alguns atributos.

Considerando que o algoritmo K-means requer que o número de clusters seja especificado, um código foi criado para identificar o número ótimo de clusters com base em três métricas (Silhouette, Davies bouldin e Calinski). Em seguida, foi feita uma validação relativa: uma tabela de mesma dimensão das tabelas de hemicorpos foi gerada com dados aleatórios, e as mesmas métricas foram calculadas e comparadas. Por fim, os atributos de cada cluster foram interpretadas por análise estatística e visualização gráfica.

## Resultados

Foram convidados todos os estudantes (n=1022) do Campus, desses, 529 se apresentaram para o estudo. Dos 529, foram excluídos os dados de 40 participantes mediante análise das informações de comorbidades (cinco hipertensos e/ou diabéticos, e/ou cardiopatas), uso de medicamentos que pudessem alterar a PS (n=18), e da idade (17 menor que 18 ou maior que 29 anos). A população final do estudo foi 489 participantes: 341 do sexo feminino (69,73%) e 148 do sexo masculino (30,27%).

### Análise descritiva

**Figura 1:**
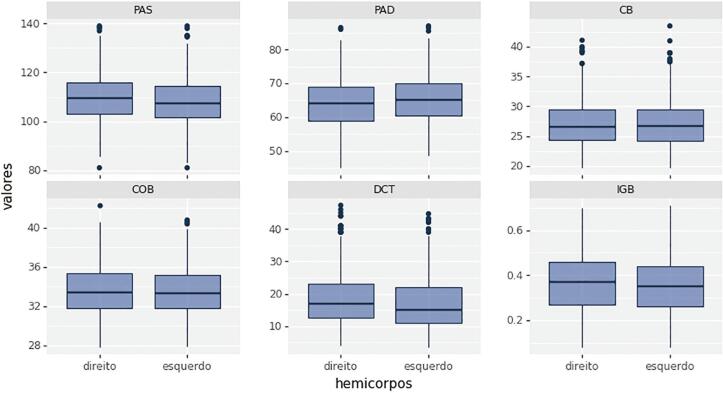
– Bloxpot das variáveis utilizadas para análise. PAS: pressão arterial sistólica; PAD: pressão arterial diastólica; COB: comprimento do braço; CB: circunferência do braço; DCT: dobras cutâneas de tríceps; IGB: índice de gordura do braço.

Os testes específicos mostraram evidências de que a PAS apresentou comportamento bastante semelhante a uma distribuição normal. Aplicando-se uma transformação logarítmica, operação muito comum em variáveis contínuas e sempre positivas, passamos a não rejeitar a hipótese nula de normalidade para a variável PAS. A PAD mostrou distribuição aproximadamente normal para braços direito (BD): p-valor = 0,323 e braço esquerdo (BE): p-valor = 0,051. Por outro lado, não há evidências de que as variáveis CB, COB, DCT, IGB e PAS, apresentem distribuição normal (p valor < 0,050) na sua escala original, para ambos os braços, conforme assimetria e *outliers* na Figura 1.

A média dos valores da PAS e as medianas de COB, DCT e IGB foram maiores no braço direito, e da PAD, no braço esquerdo (p-valor = 0,000). Não houve diferença nas medianas de CB (p-valor = 0,078) – Tabela 1.

**Tabela 1:** – Média e mediana da pressão sanguínea e medidas antropométricas por hemicorpo

Variáveis	Direito		Esquerdo		Valor de p
	Média	DP	Média	DP	
PAS	109,91	10,11	108,60	98,20	0,000
PAD	64,01	7,26	6,96	65,42	0,000
	**Mediana**	**IIQ**	**Mediana**	**IIQ**	
COB	33,40	3,60	33,33	3,40	0,000
CB	26,60	5,10	26,70	5,30	0,078
DCT	17,00	11,50	15,10	11,00	0,000
IGB	0,37	0,19	0,35	0,18	0,000

*Fonte: elaborada pelos autores. DP: desvio padrão; IIQ: intervalo interquartil; PAS: pressão arterial sistólica; PAD: pressão arterial diastólica; COB: comprimento do braço; CB: circunferência do braço; DCT: dobras cutâneas de tríceps; IGB: índice de gordura do braço.*

As variáveis COB e CB apresentaram correlação positiva com a PAS em ambos os braços. Ao contrário, o IGB apresenta correlação negativa em ambos os braços. Nos testes de correlação não foram encontrados indicativos de correlação entre PAD e as covariáveis. A Tabela 2 apresenta os valores de correlação da PAS com as principais variáveis por hemicorpos.

**Tabela 2:** – Variáveis correlacionadas com a pressão arterial sistólica por hemicorpo

Variáveis	PAS braço direito	Valor de p	PAS braço esquerdo	Valor de p
COB	0,440	0,000	0,464	0,000
CB	0,340	0,000	0,421	0,000
IGB	-0,300	0,000	-0,291	0,000

*PAS: pressão arterial sistólica; COB: comprimento do braço; CB: circunferência do braço; IGB: índice de gordura do braço.*

Considerando que a média e as medianas de PAS, COB e IGB, mas não a CB, foram diferentes entre os braços, utilizamos o total de braços (n = 978) para identificação das correlações por intervalos de CB. A aplicação da regra de Doane resultou em 15 intervalos de circunferência com espaçamento de 1,59 cm entre eles. Os cinco intervalos com maior número de ocorrências (n) foram escolhidos para testes de associação. A Tabela 3 apresenta as correlações de PAS com COB e IGB por intervalos de CB.

**Tabela 3:** – Coeficientes de correlação (r) das variáveis correlacionadas com pressão arterial sistólica por intervalo da circunferência braquial

Intervalo	CB	n	COB	Valor de p	IGB	Valor de p
**1**	**(22,87; 24,46]**	175	0,379	0,000	-0,434	0,000
**2**	**(24,46; 26,05]**	193	0,616	0,009	-0,374	0,000
**3**	**(26,05; 27,63]**	142	0,541	0,000	-0,421	0,000
**4**	**(27,63; 29,22]**	125	0,551	0,000	-0,485	0,000
**5**	**(29,22; 30,81]**	96	0,581	0,000	-0,459	0,000

*COB: comprimento do braço; CB: circunferência do braço; IGB: índice de gordura do braço.*

Observou-se correlação positiva da PAS com COB e negativa com IGB em todos os intervalos de CB. Todos os valores de correlação de IGB da Tabela 3 foram maiores do que os apresentados na Tabela 2. A partir desse resultado investigamos modelos de inferência sob a perspectiva de valores fixos de CB.

### Regressão linear

As variáveis COB, CB e IGB foram escolhidas para composição dos modelos de inferência sobre a PAS (Tabela 4). O modelo para BD apresentou estatística F=69,68 e R^2^=0,301. Já o modelo para BE apresentou estatística F=90,57 e R^2^=0,359. Há evidências de que o modelo é significativo, mas que não consegue explicar toda a variabilidade, considerando o baixo valor do coeficiente de determinação. As expressões para os modelos de BD (1) e BE (2) são:

**Tabela 4:** – Coeficientes de regressão para braço direito e esquerdo

Variável	Coeficiente		Erro padrão		t		Valor de p		0,025		0,975	
	BD	BE	BD	BE	BD	BE	BD	BE	BD	BE	BD	BE
Intercepto	59,972	53,149	6,026	5,704	9,952	9,317	0,000	0,000	48,131	41,941	71,812	64,358
CB	0,823	1,005	0,109	0,102	7,517	9,864	0,000	0,000	0,608	0,805	1,038	1,206
COB	1,017	1,006	0,171	0,164	5,952	6,129	0,000	0,000	0,681	0,684	1,352	1,329
IGB	-18,017	-16,224	3,411	3,182	-5,282	-5,098	0,000	0,000	-24,719	-22,477	-11,314	-9,971

*BD: braço direito; BE: braço esquerdo; CB: circunferência do braço; COB: comprimento do braço; IGB: índice de gordura do braço.*


 PAS =59,97+0,82CB+1,01COB−18,01/GB (1)

PAS =53,15+1,00CB+1,01COB−16,22IGB (2)

### Clustering

A exploração dos dados pela clusterização apontou a divisão dos dados em dois clusters como melhor solução, a partir das métricas pré-estabelecidas. A Tabela 5 apresenta os valores da média para cada *feature* para ambos os braços. Os grupos identificados pela clusterização mostram que em ambos os braços, os maiores valores de PAS (cluster 0) foram encontrados nos braços com maiores COB e menores CB, DCT e IGB.

**Tabela 5:** – Média das variáveis clusterizadas por braço direito e esquerdo

Braço	Cluster	COB	CB	DCT	IGB	PAS	PAD
Direto	0	34,30	26,23	13,38	0,30	111,83	64,09
	1	32,66	28,87	27,08	0,50	106,71	63,88
Esquerdo	0	34,18	26,18	11,78	0,26	110,08	65,17
	1	32,58	28,74	25,60	0,48	106,29	65,81

*Fonte: elaborada pelos autores. COB: comprimento do braço; CB: circunferência do braço; DCT: dobras cutâneas de tríceps; IGB: índice de gordura do braço; PAS: pressão arterial sistólica; PAD: pressão arterial diastólica.*

## Discussão

Foram incluídas nas análises apenas as variáveis que tiveram correlação estatisticamente significativas com PAS e/ou PAD: DCT, IGB, COB e CB. Outras variáveis como IMC, raça e idade não tiveram correlação estatisticamente significativas.

Embora comparações das medidas e estimativas antropométricas entre os hemicorpos têm sido objeto de pesquisas recentes, de modo geral, as investigações não incluem medidas de PS nas análises, e seus objetivos estão relacionados à avaliação de treinamento, força e movimento muscular. Todavia, estudos têm mostrado assimetrias entre os braços como maior massa,^25^ e maior dimensão, peso e espessura do úmero^26^ do braço dominante (direito) em comparação ao esquerdo. Por outro lado, Macedo et al.^27^ compararam DCT entre os lados dominante e não dominante em grupos com comprometimento unilateral e controle. Os autores encontraram diferenças estatisticamente significativas inclusive no grupo controle e argumentam que as medidas foram padronizadas há muitos anos e tomadas na metade direita do corpo. Outras perspectivas discutem a relação da gordura central com as doenças crônicas não transmissíveis.

O resultado dos grupos identificados pela clusterização corroboraram as correlações identificadas entre ambos os braços. Conforme o modelo para braço direito (equação 1), fixando um valor qualquer para CB e COB, a mensuração indireta da PAS resultará na redução média de 1,80mmHg a cada acréscimo de 10% no IGB (Figura Central). No braço esquerdo, a redução média será de 1,62mmHg. Dessa forma, em nossa amostra, uma variação no IGB de 8% a 70% no braço direito levará em uma redução na PAS de 11,17mmHg em média. Por exemplo, para uma pessoa com CB = 27,22cm, COB = 33,68cm, IGB = 37%, e PAS = 109,64mmHg, (valores relativos às médias da população do estudo) se IGB for 50%, a PAS será 107,30 mmHg, em média. Se o valor de IGB aumentar para 70%, a PAS média será 103,70 mmHg. No exemplo, a variação da PAS em função do IGB foi maior que 5mmHg. Essa variação pode ser determinante no diagnóstico e/ou estadiamento da HAS e expor um indivíduo a consequências já discutidas.

O IGB estima a reserva de gordura do braço contida na área de mensuração indireta da PS. A correlação negativa da PAS com o IGB pode ser interpretada em função da diferença das densidades dos tecidos humanos, mais especificamente do músculo e da gordura. A densidade do músculo esquelético de mamífero é estimada em 1,06 Kg/L e do tecido adiposo em 0,92 Kg/L.^28-30^ A menor densidade na área de medida da PS exige menos força para a compressão da artéria braquial e, dessa forma, subestimam-se os valores pressóricos. Ou seja, a MIPS no braço com maior percentual de gordura implicará na leitura mais baixa que no braço com menos gordura, porque a gordura é menos densa e oferece menos resistência para compressão. No mesmo sentido, a leitura da PS poderá ser mais alta no braço com menos gordura.

Conforme já mencionado, não encontramos publicações em revistas específicas da área da saúde que investigassem possíveis relações entre composição do braço e a MIPS. Entretanto, a MIPS tem sido objeto de discussão na engenharia, especialmente a biomédica. Análises matemáticas performadas com modelos de elementos finitos (EF) têm sido desenvolvidos na busca de soluções para a redução da imprecisão do procedimento.^31-33^

Lan et al.^34^ desenvolveu um interessante modelo 3D para investigação do efeito das propriedades mecânicas do tecido sobre a medida da PS. Os autores concluíram que a PS é superestimada em cerca de 5% nos idosos, em função da compressibilidade de tecidos moles de 0,4, e subestimada nas crianças em cerca de 5% em função da maior compressibilidade (0,49), e que a variação da rigidez da artéria braquial não afeta a precisão da medida oscilométrica da PA. Esta conclusão, no entanto, não foi corroborada por Liang et al.^35^ Nesse sentido, salienta-se que indivíduos obesos frequentemente apresentam maior rigidez nas artérias.

O Deng e Liang^32^ propuseram um modelo para simular a distribuição de estresse nos tecidos do braço sob um manguito. Os autores mencionam ajustes no modelo proposto por Lan et al.^34^ e concluíram que a magnitude da pressão do manguito tem pouca influência na eficiência da transmissão da pressão nos tecidos do braço, confirmando parcialmente a confiabilidade da medição não invasiva da pressão arterial, baseada tanto em indivíduos normotensos quanto em hipertensos. Os autores também mencionaram que o espessamento da camada de gordura subcutânea em pacientes obesos amorteceu significativamente a transmissão da pressão do manguito nos tecidos do braço, o que explicaria a superestimativa da pressão arterial nestes indivíduos. Entretanto, na nossa perspectiva, o aumento do tecido adiposo encontra limite nos demais tecidos como os músculos, o osso e na capacidade elástica da pele. Dessa forma, uma maior concentração de gordura no espaço limitado aumenta a densidade, consequentemente aumenta o valor da PS na MIPS.

Todavia, modelar um braço real parece uma tarefa complexa, o que impõe maiores limitações aos estudos na investigação da influência da composição do braço sobre a MIPS. A limitação do nosso trabalho foi o uso de medidas antropométricas para estimativa da composição corporal do braço. Entretanto, todas as medidas foram realizadas por um único pesquisador, o que contribui com a redução de eventuais imprecisões relacionadas à técnica. Além disso, medidas antropométricas de obesidade abdominal não foram coletadas no protocolo e as associações estudadas não foram ajustadas para essas variáveis. Por outro lado, esta pesquisa apresenta uma incógnita ainda não investigada na mensuração da PS em estudo com humanos.

Investigações que relacionam variáveis distintas e doenças, por exemplo a diferença da PS entre os braços e as DCV,^36^ são frequentes. Entretanto, não encontramos estudos recentes que discutam diferenças da PS entre os braços em indivíduos jovens saudáveis. Assim, sugerimos para pesquisas futuras as seguintes questões: pessoas com maior área gordurosa do braço, como as mulheres, têm a mensuração da PS subestimada? Será necessário um algoritmo para correção dos valores pressóricos em função da diferença das frações de gordura e músculo?

## Conclusão

Na MIPS, os braços com maior IGB têm valores subestimados para PAS. A média dos valores pressóricos e das medidas antropométricas dos adultos jovens saudáveis foi diferente entre os hemicorpos. Não foram encontrados estudos semelhantes para comparação com os resultados.
