# Supplementary material for: Influência da Gordura do Braço sobre Medida Indireta da Pressão Sanguínea: Uma Abordagem Estatística e de Machine Learning
Source: Arq Bras Cardiol. 2023 May 5;120(5):e20220484. [Article in Portuguese] doi: 10.36660/abc.20220484 (PMC10348388; doi:10.36660/abc.20220484)
Supplement: Translation

# Translation: Influence of Arm Fat on the Indirect Measurement of Blood Pressure: A Statistical and Machine Learning Approach

**Authors:** Paôla de Oliveira Souza, José Maria Parente de Oliveira, Letícia Helena Januário

**Affiliations:** 1 Brazil Instituto Federal de Minas Gerais, Ibirité, MG – Brazil; 2 Brazil Instituto Tecnológico de Aeronáutica, São José dos Campos, SP – Brazil; 3 Brazil Universidade Federal de São João del-Rei, Divinópolis, SP – Brazil

**Keywords:** Arterial Pressure, Regression Analysis, Cluster Analysis, Arm

## Abstract

**Background:**

The indirect measurement of blood pressure (BP) is known to be influenced by many factors such as the technique, observer, and equipment; however, the influence of arm composition has not been investigated yet.

**Objective:**

To identify the influence of arm fat on the indirect measurement of blood pressure using statistical inference and machine learning models.

**Methods:**

Cross-sectional study, with 489 healthy young adults aged from 18 to 29 years old. Measurements of arm length (AL), arm circumference (AC) and arm fat index (AFI) were taken. BP was measured in both arms simultaneously. Data were processed using Python 3.0 and its specific packages for descriptive analysis, regression and cluster analysis. Significance levels: 5% for all calculations.

**Results:**

BP and anthropometric measurements were different between the hemi-bodies. In the right arm, systolic blood pressure (SBP), AL and AFI were higher, while AC was similar compared with the left arm. AL and AC showed positive correlation with SBP. According to the regression model, for a fixed value of AC and AL, SBP readings could be reduced by a mean of 1.80 mmHg in the right arm, and 1.62 mmHg in the left arm for every 10% increase in AFI. Clustering analysis corroborated the regression results.

**Conclusion:**

There was a significant influence of AFI on BP readings. SBP had a positive correlation with AL and AC, and a negative correlation with AFI, suggesting the need for further investigations on the relationship between BP and percentages of arm muscle and fat.

## Introduction

An accurate measurement of blood pressure (BP) is essential for an appropriate diagnosis and treatment of systemic arterial hypertension (SAH). Since most SAH patients are asymptomatic, the diagnosis is usually made in routine consultations at the medical office or at the primary care. Despite technological advances in the treatment of target organ lesions, the identification and classification of SAH, as well as treatment thresholds and goals, are still based on the indirect measurement of blood pressure (IMBP).^1^

Standardization of IMBP techniques, recommended in several guidelines, highlights the selection of cuffs, that must be proportional to the arm circumference (AC).^2-8^ However, for the same AC, there may be different proportions of arm fat and muscle. These structures have different densities and thicknesses, which may require higher or lower pressure on the limb for artery compression and constitute an important cause of inaccuracy in IMBP. Errors and inaccuracies in this procedure could cause either unnecessary prescription or a failure to provide urgent treatment.^9^

The possible inaccuracy of IMBP as a function of different percentages of arm fat and muscle in individuals with the same AC was not found in the specialized literature consulted. The identification of this variable could result in a more accurate estimation of BP values measured by indirect methods.

On the other hand, the combination of traditional statistics with machine learning (ML) in medical studies is continuously rising. The use of ML has important advantages in prediction performance, in the determination of properties and possible correlations of attributes in a data set, and the identification of undiscovered subpopulations and specific prognostics.^10,11^

The objective of this study was to identify the influence of arm fat on IMBP in healthy young adults, using models of statistical inference and ML in a clusterization approach.

BP measurement is a widely spread procedure, performed by many health professionals, and also by laypeople. For this reason, it may be characterized as a “trivial” practice and performed carelessly and/or by bad habits acquired in the beginning of professional training.^12^ However, the accuracy of the procedure requires specific competencies and attention to small details, something frequently seen, even when conducted by professionals.^1,13^

Measurement of BP is usually made indirectly. According to the main international and national guidelines, the technique requires care of the individual having the BP measured, the professional, the environment, and the equipment used.^2,5,7,8,14-19^

Among recommendations, an appropriate cuff size – length of 80-100% and 40% of AC.^7,14,17,18^The use of a cuff with dimensions larger or smaller than the ideal leads to underestimation and overestimation of BP values respectively.^9,20^

Although the auscultation method with an aneroid manometer is still widely used in Brazil, there has been an increasing use of automated oscillometric devices, which has reduced the errors related to professional observation.^21^Nonetheless, regardless of the equipment used, BP measurement is determinant in SAH diagnosis, with a large margin of variation.

## Materials and Methods

This was a cross-sectional, analytical, descriptive study, with data collected at the Federal University of São João del-Rei in Divinópolis, Brazil. The study population was composed of undergraduate students aged between 18 and 29 years. This was a convenience sample due to limited financial resources and geo-economic diversity in the population of healthy young adults at the university in question. Data were collected from August 2017 to January 2018, by means of questionnaires, and measurements of triceps skinfold (TS) thickness, AC, arm length (AL), and BP.

### Anthropometric measurements

TS thickness was measured in millimeters, and AC and AL in centimeters in both arms. All measurements were made with certified, validated, and properly calibrated equipment according to the techniques described by the NHANES.^22^ Three measurements of each variable were made, and the mean was used for analysis. The anthropometric measurements were used for calculation of the arm fat index (AFI) according to Frisancho,^23^ using the equation:


$$AFI=\frac{AFA}{TAA}\times 100$$

Where AFA is the arm fat area (cm^2^) and TAA is the total arm area (cm^2^) whose formulas are, respectively:^23^


$$\begin{array}{c} AFA=\frac{2\times TSA\times CB-\pi {TS}^{2}}{4}\\ TAA=\frac{{AC}^{2}}{4\pi } \end{array}$$

### Blood pressure

Measurements of BP strictly followed national and international guidelines,^1,4^and were taken in a private, calm, laid-back environment, under good lighting. Researchers tried to establish an effective interpersonal relationship with the subjects, with welcoming posture and attitudes, as recommended by Kohlmann and Kohlmann.^24^It was confirmed that participants had not smoked, exercised or consumed alcoholic beverages, coffee, or food in the 60 minutes before, and they were asked to empty their bladders before measurements. There was a rest period of at least 10 minutes before the reading was taken. Participants sat relaxed and leaning back in a height-adjustable, comfortable armchair with arm and leg rest, with legs uncrossed, and were instructed not to talk during the measurements. The arms were positioned at the height of the heart, with the palm of the hand turned up. Conical cuffs were used, adjusted to the circumference of the arm, and positioned from 2 to 3 cm above the antecubital fossa, with the midline of the bladder placed over the brachial artery.^1,18^Three measurements were taken in both arms simultaneously, with one-minute intervals between them, using a validated, calibrated oscillometric device by Omron. Simultaneity of the measurements was assured by the filming of displays. For the analysis, an average of the last two measurements was used, in accordance with Leung.^18^

### Data analysis

Data were imported and managed in the JupyterLab environment (version 2.2.6) and analyzed using the Python 3.0 language and the Pandas, NumPy, MatplotLib, Statsmodels, Seaborn, Plotnine, Sci-Py and Sklearn packages. The dataset used and the notebooks with procedures and codes (descriptive analysis, regression, and clustering) are available in a GitHub repository (https://github.com/paolaosouza/ blood_preassure_analysis) for consultation and reproduction purposes by other researchers.

### Statistical analysis

Data were arranged in a table, and those variables that showed statistically significant correlation with systolic blood pressure (SBP) and/or diastolic blood pressure (DBP) were selected: AL, AC, TS, and AFI. A column of circumference intervals was created using the Doane’s rule. Data were also divided into two other tables, one with measurements of the right arm, and another one with measurements of the left arm.

The significance level was set as 5% (p-value < 0.05) for all tests in the descriptive analysis. The Shapiro-Wilk test was used to test the assumption of normality of continuous variables. Continuous variables with normal distribution were described as mean and standard deviation, while those without normal distribution were described as median and interquartile range. The means of continuous variables with normal distribution were compared by body side, using the paired T-test and for the medians were compared by the non-parametric Wilcoxon test, since the groups are dependents. The association between continuous variables was assessed by Spearman’s correlation coefficient (r).

A multiple linear regression model was adjusted for each hemi-body to make inferences on the relationship between SBP and the covariables. No regression model was adjusted for DBP, as there was no evidence of its association with covariables. Highly correlated variables were discarded to maintain a lean model and avoid multicollinearity, prioritizing variables compatible with the study’s aim. Then, one model was adjusted for each hemi-body and another one for data from both arms considering the variable “arm” as a random effect. The quality of adjustment was evaluated by the Akaike information criterion (AIC), and models were validated by residue analysis. The normality assumption was checked with the Q-Q plot and the Shapiro-Wilk test; independence of residues was checked by ACF plot and the Durbin-Watson statistic; and homoscedasticity by plotting techniques, by analysis of residuals *versus* covariables.

### Machine learning (ML) analysis

ML models were used to add important information to the statistical analyses.^9^An unsupervised clustering approach was used to explore the data in a way “free from hypotheses” to try to identify underlying patterns of BP and anthropometric measures. The K-means clusterization algorithm was chosen because it is relatively simple, computationally fast to implement, and provides reasonable results to most problems.^10^

The K-means algorithm was applied to the group of data of left and right arms separately. Initially, results of all attributes were normalized to the same interval to avoid prioritizing some attributes only.

Considering that the K-means algorithm requires a specified cluster number, a code was created to determine the optimal number of clusters based on three metrics (Silhouette, Davies bouldin e Calinski). Subsequently, a relative validation was performed: a table of random data, with the same dimensions as the one of hemi-body data was generated, and the same metrics were calculated and compared. Finally, the attributes of each cluster were interpreted by statistical analysis and graphical visualization.

## Results

All students at the campus were invited (n=1022), and 529 of them presented themselves for the study. Data of 40 participants were excluded after analysis of data concerning health (five had high blood pressure and/or diabetes and/or cardiopathy), use of medicine that could affect BP (n=18), and age (17 aged lower than 18 or higher than 29 years). The final group of participants was 489: 341 women (69.73%) and 148 men (30.27%).

### Descriptive analysis

The specific tests showed evidence that the SBP showed a behavior that was similar to that of a normal distribution. After applying the logarithmic transformation, which is very common in continuous and always positive variables, we started to accept the null hypothesis of normality for the SBP variable. The DBP showed an approximately normal distribution for the right arm (RA): p-value = 0.323 and left arm (LA): p-value = 0.051. On the other hand, there was no evidence of normal distribution of the variables AC, AL, TS, AFI and SBP (p value < 0.050) in their original scale, for both arms, according to asymmetry and outliers in Figure 1.

**Figure 1:** – Bloxpot of variables used for analysis. SBP: systolic blood pressure; DBP: diastolic blood pressure; AL: arm length; AC: arm circumference; TS: triceps skinfold; AFI: arm fat index.

Mean SBP values and median values of AL, TS, and AFI were higher on the right arm, and DBP was higher in the left arm (p-value = 0.000). There was no difference in median AC values (p-value = 0.078; (Table 1).

**Table 1:** – Mean and median blood pressure and anthropometric measurements by hemi-body (right/left)

Variables	Right		Left		p-value
	Average	SD	Average	SD	
SBP	109.91	10.11	108.60	98.20	0.000
DBP	64.01	7.26	6.96	65.42	0.000
	**Median**	**IR**	**Median**	**IQR**	
AL	33,40	3,60	33,33	3,40	0.000
AC	26,60	5,10	26,70	5,30	0.078
TS	17,00	11,50	15,10	11,00	0.000
AFI	0,37	0,19	0,35	0,18	0.000

*SD: standard deviation; IQR: interquartile range; SBP: systolic blood pressure; DBP: diastolic blood pressure; AL: arm length; AC: arm circumference; TS: triceps skinfold; AFI: arm fat index.*

Both AL and AC showed a positive correlation with SBP in both arms. On the contrary, AFI showed a negative correlation in both arms. In the correlation tests, there was no indication of correlation between DBP and covariables. Table 2 presents values of correlation of SBP with the main variables by body side.

**Table 2:** – Variables correlated with systolic blood pressure by hemi-body

Variables	Right arm SBP	P-value	Left arm SBP	p-value
AL	0.440	0.000	0.464	0.000
AC	0.340	0.000	0.421	0.000
AFI	-0.300	0.000	-0.291	0.000

*Source: made by the authors. SBP: systolic blood pressure; AL: arm length; AC: arm circumference; AFI: arm fat index.*

Considering that mean and median SBP, AL, and AFI values, but not AC, were different between arms, we used the total number of arms (n = 978) to identify correlations by AC intervals. Application of Doane’s rule resulted in 15 intervals of AC with a spacing of 1.59 cm between them. The five intervals with the highest number of occurrences (n) were chosen for association tests. Table 3 presents correlations of SBP with AL and AFI by intervals of AC.

**Table 3:** – Correlation coefficients(r) of the variables correlated with systolic blood pressure by arm circumference intervals

Interval	AC	n	AL	p-value	AFI	p-value
**1**	**(22.87, 24.46]**	175	0.379	0.000	-0.434	0.000
**2**	**(24.46, 26.05]**	193	0.616	0.009	-0.374	0.000
**3**	**(26.05, 27.63]**	142	0.541	0.000	-0.421	0.000
**4**	**(27.63, 29.22]**	125	0.551	0.000	-0.485	0.000
**5**	**(29.22, 30.81]**	96	0.581	0.000	-0.459	0.000

*AL: arm length; AC: arm circumference; AFI: arm fat index.*

There was a positive correlation of SBP with AL, and a negative correlation with IGB in all intervals of AC. All AFI correlation values in Table 3 were higher than the ones in Table 2. From these results, we investigated inference models from fixed AC values.

### Linear regression

The variables AL, AC, and AFI were chosen for the inference models of SBP (Table 4). The model for RA had an F-statistic of 90.57 and R^2^=0.359. There is evidence that the model is significant, but it does not explain all variability, considering the low coefficient of determination. The equations proposed for the models of the RA and the LA are, respectively:

**Table 4:** – Regression coefficients for the right and left arms

Variable	Coefficient		Standard error		t		p-value		0.025		0.975	
	RA	LA	RA	LA	RA	LA	RA	LA	RA	LA	RA	LA
Intercept	59.972	53.149	6.026	5.704	9.952	9.317	0.000	0.000	48.131	41.941	71.812	64.358
AC	0.823	1.005	0.109	0.102	7.517	9.864	0.000	0.000	0.608	0.805	1.038	1.206
AL	1.017	1.006	0.171	0.164	5.952	6.129	0.000	0.000	0.681	0.684	1.352	1.329
AFI	-18.017	-16.224	3.411	3.182	-5.282	-5.098	0.000	0.000	-24.719	-22.477	-11.314	-9.971

*RA: right arm; LA: left arm; AC: arm circumference; AL: arm length; AFI: arm fat index.*


SBP=59.97+0.82AC+1.01AL−18.01AFI (1)

SBP=53.15+1.00AC+1.01AL−16.22AFI (2)

### Clustering

The analysis of data by the clustering method indicated the division of data into two clusters as the best solution, based on the pre-established metrics. Table 5 presents mean values of each feature for both arms. The groups identified by clustering showed that in both arms, higher values of SBP (cluster 0) were found in arms with higher AL and lower AC, TS, and AFI.

**Table 5:** – Mean of cluster variables by arm (right/left)

Arm	Cluster	AL	AC	TS	AFI	SBP	DBP
Right	0	34.30	26.23	13.38	0.30	111.83	64.09
	1	32.66	28.87	27.08	0.50	106.71	63.88
Left	0	34.18	26.18	11.78	0.26	110.08	65.17
	1	32.58	28.74	25.60	0.48	106.29	65.81

*AL: arm length; AC: arm circumference; TS: triceps skinfold; AFI: arm fat index; SBP: systolic blood pressure; DBP: diastolic blood pressure.*

## Discussion

The variables that showed statistically significant correlation with SBP and/or DBP – TS, AFI, AL and AC – were included in the analysis. Other variables, such as body mass index, ethnicity, and age had no statistically significant correlation with SBP or DBP.

Although comparisons of anthropometric estimations and measurements between hemi-bodies have been the object of recent research, most investigations do not include BP measurements in the analysis, and their objectives are related to the evaluation of training, and muscle strength and movement. However, studies have shown an asymmetry between the arms, such as greater mass,^25^ and bigger, heavier, and thicker humerus in the dominant (right) arm^26^ compared with the left arm than the left one.^26^ On the other hand, Macedo et al.^27^compared TS thickness between dominant and non-dominant sides in individuals with cerebral palsy and spastic hemiplegia and control subjects. The authors found statistically significant differences that were even in the control group and argued that measurements were standardized years ago and taken from the right side of the body. Other reports have discussed the relationship between central fat and noncommunicable chronic diseases.

Results of the groups identified by clustering corroborate the correlations found between both arms. In accordance with the model for the right arm (equation 1), for a fixed value of AC or AL, the indirect measurement of SBP will result in a mean reduction of 1.80mmHg to each 10% increase in AFI (Figure 2). In the left arm, the mean reduction will be of 1.62mmHg. Thus, in our sample, for a variation in AFI from 8% to 70%, there will be a reduction of 11.17mmHf in SBP. For example, for a person with AC of 27.22cm, AL of 33.68 cm, AFI of 37%, and SBP of 109.64mmHg (values relative to the means of the study population) if AFI is 50%, mean SBP will be of 107.30 mmHg. If the value of AFI increases to 70%, mean SBP will be 103.70 mmHg. In the example, the variation of SBP in function of AFI was higher than 5mmHg. This variation can be determinant in the diagnostic and/or staging of SAH and exposing an individual to consequences already discussed.

The AFI estimates the amount of fat stored in the arm in the indirect measurement area of BP. The negative correlation of SBP with AFI may be explained by differences in human tissues, more specifically muscle and fat. The density of mammalian skeletal muscle is estimated in 1.06 Kg/L and of the adipose tissue in 0.92 Kg/L.^28-30^ The lower density in the BP measurement area demands lower compression force of the brachial artery, resulting to underestimation of pressure values. Therefore, the IMBP on the arm with a higher fat percentage will imply a lower reading than in the arm with less fat, because fat is less dense and offers less resistance for compression. In the same sense, BP reading may be higher in individuals with less body fat.

As previously mentioned, we found no publications in health journals that investigated possible relationships between arm composition and IMBP. However, IMBP has been subject to discussion in engineering, especially in the field of biomedicine. Mathematic analysis performed with finite element (FE) models have been developed in the attempt to reduce the inaccuracy of the procedure.^31-33^

Lan et al.^34^ developed an interesting 3D model to investigate the effect of mechanical properties of the tissue on BP measurement. The authors concluded that BP is overestimated in about 5% of the elderly, due to the compressibility of soft tissues of 0.4, and overestimated in children in nearly 5% due to the higher compressibility (0.49). Also, they concluded that the variation in brachial artery rigidity did not affect the precision of oscillometric measurements of BP. This conclusion was not corroborated by Liang et al.^35^ In this sense, obese individuals frequently have higher arterial rigidity.

Deng and Liang^32^ proposed a model to simulate the distribution of stress in arm tissues under a cuff. The authors mentioned adjusts on the model proposed by Lan et al.^34^ and concluded that the magnitude of the cuff pressure has a low influence on the efficiency of pressure transmission in arm tissues, partially confirming the reliability of non-invasive BP measurement, based on both hypertensive and normal BP individuals. The authors also mentioned that thickening of the subcutaneous fat layer in obese patients significantly minimized the transmission of the cuff pressure throughout the arm tissues, which would explain the overestimation of arterial pressure in these individuals. However, in our perspective, the increase of fat tissue is limited by other tissues, such as muscle, bone, and skin elasticity. Therefore, a higher concentration of fat in a limited space increases the density, increasing BP values in IMBP.

However, modeling a real arm seems like a complex task, imposing higher limitations to studies in the investigation of the influence of the arm composition on IMBP. The limitation of this work was the use of anthropometric measurements for the estimation of arm composition. However, all measurements were made by the same investigator, contributing to the reduction of eventual imprecisions related to the technique. Besides, anthropometric measurements of abdominal obesity were not collected in the protocol and the studied associations were not adjusted for these variables. On the other hand, this study points out an aspect of BP measurement that is still poorly explored in studies with humans.

Although investigations of the association of different variables with diseases, for example, of interarm differences of BP with CVD^36^ are common, we found no recent studies discussing differences of BP between arms in healthy young individuals. Thus, we suggest that future research should address the following questions: are BP measurements underestimated in people with a greater arm fat area, such as women? Would an algorithm for correction of BP values as a function of differences in the fat-to-muscle ratios be necessary?

## Conclusion

In IMBP, SBP measurements are underestimated in arms with higher AFI. Measurements of BP and anthropometric measurements were different between the sides of the body in healthy young adults. No similar studies were found to compare the results.
